# Supplementary material for: Potent Apoptosis Induction by a Novel Trispecific B7-H3xCD16xTIGIT 2+1 Common Light Chain Natural Killer Cell Engager
Source: Molecules. 2024 Mar 4;29(5):1140. doi: 10.3390/molecules29051140 (PMC10935230; doi:10.3390/molecules29051140)
Supplement: Supplementary file 1 [file molecules-29-01140-s001.zip › molecules-2774928-supplementary.pdf]

Supplementary information

Potent Apoptosis Induction by a Novel Trispecific B7-H3xCD16xTIGIT 2+1 Common Light Chain Natural Killer Cell Engager

Michael Ulitzka[1], Julia Harwardt[1], Britta Lipinski[1], Hue Tran[1], Björn Hock[1], Harald Kolmar[1,2] *

1 Institute for Organic Chemistry and Biochemistry, Technical University of Darmstadt, Peter-Grünberg-Str. 4, 64287 Darmstadt, Germany
2 Centre of Synthetic Biology, Technical University of Darmstadt

* Correspondence: harald.kolmar@tu-darmstadt.de

1. Screening of anti-B7-H3 Fab

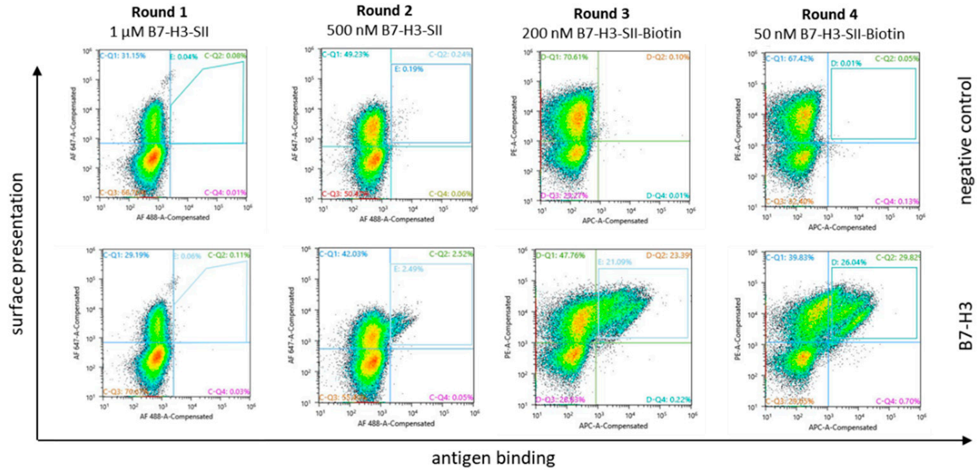

Figure S1. FACS plots of the anti-B7-H3 screening campaign.

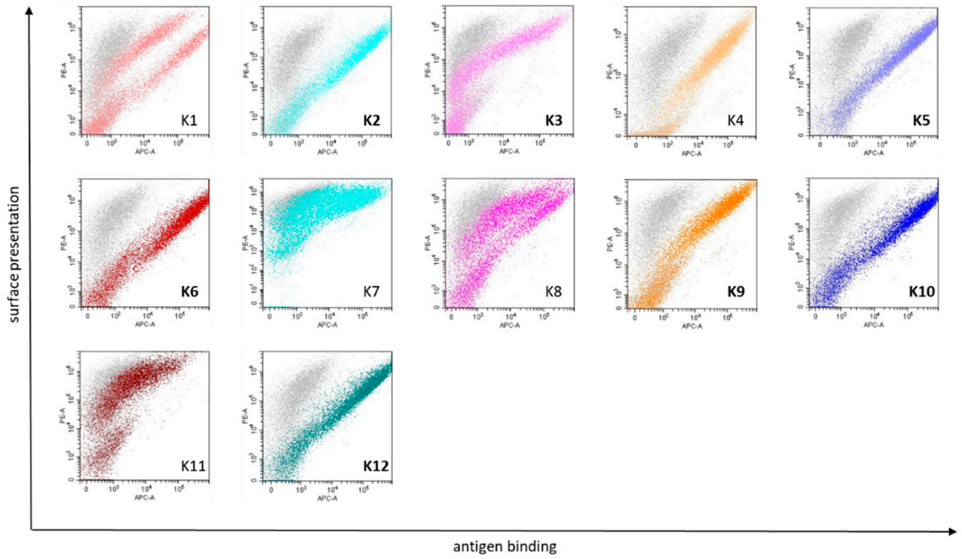

Figure S2. Single clone analysis of anti-B7-H3 Fabs.

2. Screening of anti-TIGIT Fab

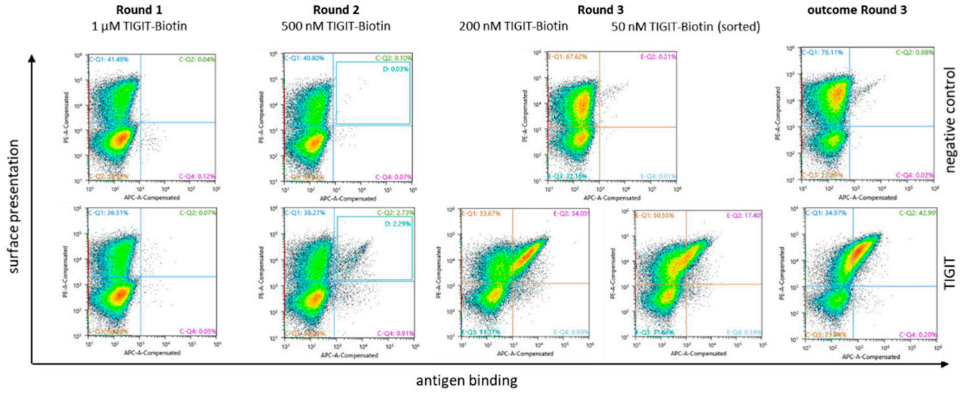

Figure S3. FACS plots of the anti-TIGIT screening campaign.

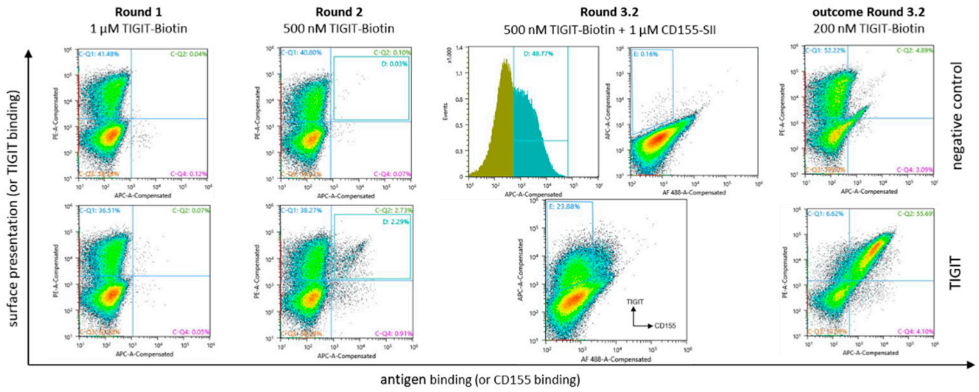

Figure S4. FACS plots of the anti-TIGIT screening campaign focusing on blocking antibodies.

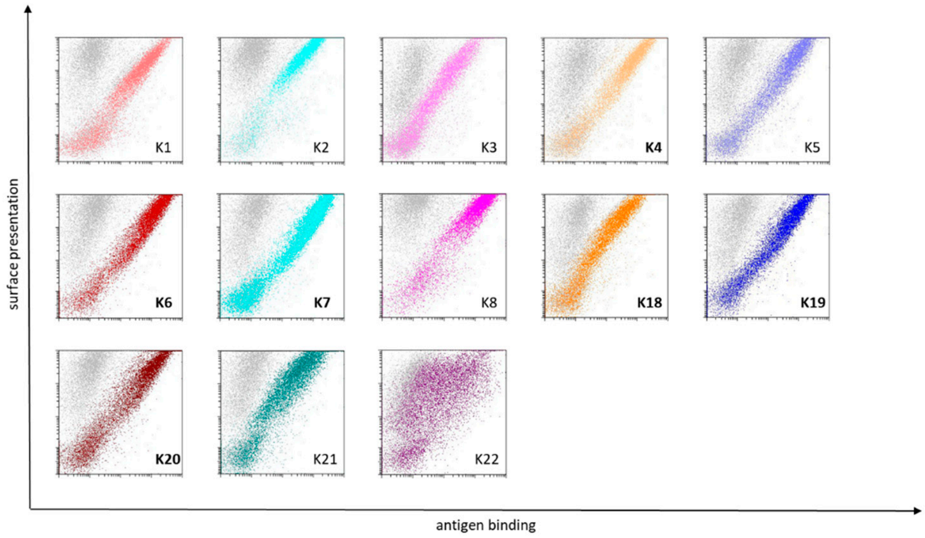

Figure S5. Single clone analysis of anti-TIGIT Fabs derived from both screening campaigns.

3. Killing assay

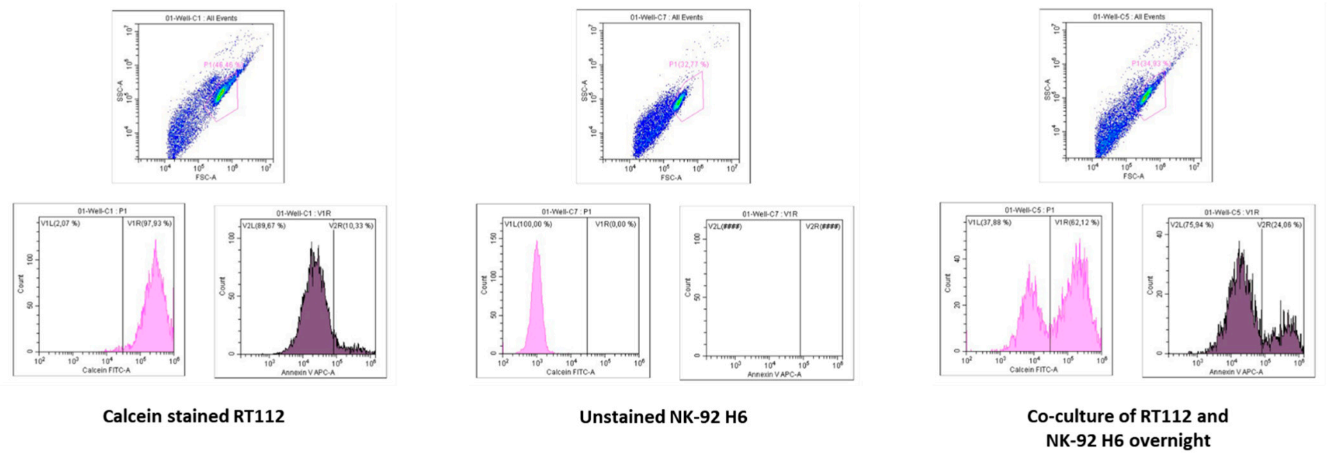

Figure S6. Gating strategy used for the analysis of apoptosis induction in the NK-92 H6-based assay.

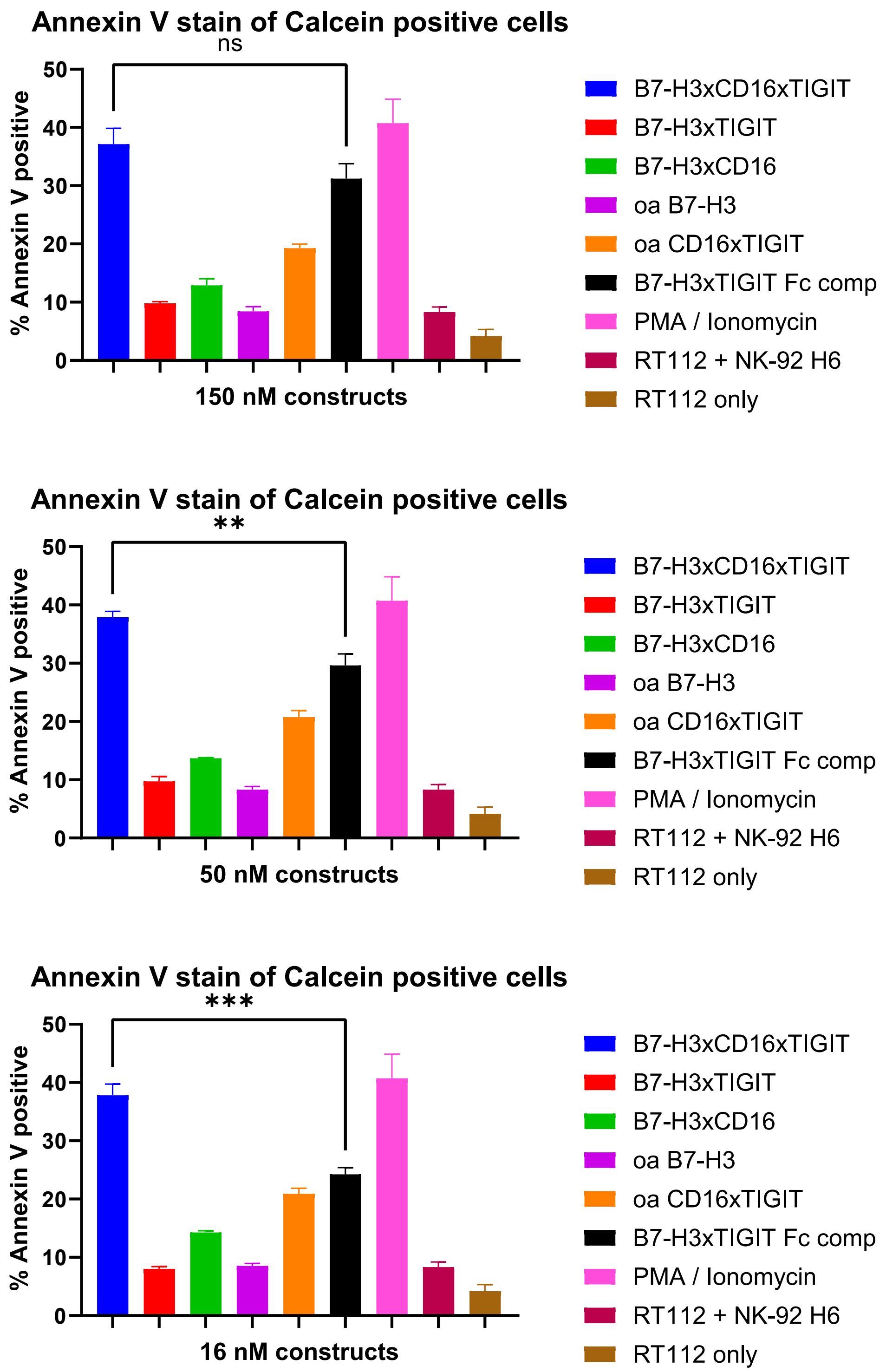

Figure S7. Apoptosis induction in RT112 cells by different antibody constructs in the NK-92 H6-based assay.
